# Pandemic Surveillance and Racialized Subpopulations: Mitigating *Vulnerabilities* in COVID-19 Apps

**DOI:** 10.1007/s11673-020-10034-7

**Published:** 2020-08-25

**Authors:** Tereza Hendl, Ryoa Chung, Verina Wild

**Affiliations:** 1 Institute of Ethics, History and Theory of Medicine, Ludwig-Maximilians-University in Munich, Lessingstr. 2, 80336 Munich, Germany; 2 Department of Philosophy, Université de Montréal, C.P. 6128, succ. Centre-Ville, Montréal, Québec H3C 3J7 Canada

**Keywords:** Pandemic disease surveillance, Digital health technologies, COVID-19, Solidarity COVID-19 apps, Vulnerability, Racial inequality, Racialized subpopulations, Justice, Equity

## Abstract

Debates about effective responses to the COVID-19 pandemic have emphasized the paramount importance of digital tracing technology in suppressing the disease. So far, discussions about the ethics of this technology have focused on privacy concerns, efficacy, and uptake. However, important issues regarding power imbalances and vulnerability also warrant attention. As demonstrated in other forms of digital surveillance, vulnerable subpopulations pay a higher price for surveillance measures. There is reason to worry that some types of COVID-19 technology might lead to the employment of disproportionate profiling, policing, and criminalization of marginalized groups. It is, thus, of crucial importance to interrogate vulnerability in COVID-19 apps and ensure that the development, implementation, and data use of this surveillance technology avoids exacerbating vulnerability and the risk of harm to surveilled subpopulations, while maintaining the benefits of data collection across the whole population. This paper outlines the major challenges and a set of values that should be taken into account when implementing disease surveillance technology in the pandemic response.

## Introduction

Debates about effective responses to the COVID-19 pandemic have emphasized the importance of digital technology in releasing lockdowns while further suppressing the disease (Schaefer and Ballantyne 2020). Apps have played a crucial role in COVID-19 response in countries that have successfully bent the infection curve, such as Taiwan (Ienca and Vayena 2020), in combination with other measures, including social distancing and testing. Many countries have since been developing and implementing mobile tracing systems to tackle the ongoing COVID-19 pandemic, amid discussions about the social impact of these technologies (Lucivero et al. 2020; Parker et al. 2020; UNESCO 2020).

So far, debates about the ethics of COVID-19 apps have been largely preoccupied with privacy concerns, transparency and open source code, or data security (Ienca and Vayena 2020; Cho et al. 2020; Bock et al. 2020). Many have argued that COVID-19 data ought to be handled with respect for privacy and confidentiality (Ienca and Vayena 2020; Parker et al. 2020; UNESCO 2020). This is an important debate, as privacy rights safeguard many liberties (Ienca and Vayena 2020). For example, the UNESCO *Statement on COVID-19: Ethical Considerations from a Global Perspective* ( 2020, 4) emphasizes that values of privacy and autonomy should be “carefully balanced with values ​​of safety and security.” Respect for privacy in COVID-19 responses is also important as a failure to comply with requirements of confidentiality can undermine public trust and deter people from following public-health recommendations and negatively affect health outcomes (Ienca and Vayena 2020). Hence, some suggest (Parker et al. 2020; CIFAR 2020) that, ideally, data provided to apps should be voluntarily self-reported and anonymized.

The concern about vulnerability is crucial as crises such as pandemics and their aftermaths typically do not affect individuals equally (Farmer 2009; Uscher-Pines et al. 2007; Oakes and Kaufman 2006). The UNESCO (2020, 2) statement on COVID-19 recognizes that in times of pandemic “vulnerable individuals become even more vulnerable.” Preliminary evidence has shown that vulnerable groups such as racialized people are among those most negatively affected by the COVID-19 pandemic (Office for National Statistics 2020; Covid Tracking Project 2020). Importantly, scholars of vulnerability (Enarson 2012; Hunt et al. 2015; Durocher et al. 2016) have argued that responses to disastrous events which do not carefully interrogate vulnerability in the target population risk exacerbating the vulnerability of already marginalized subpopulations.

In order to unpack such exacerbated vulnerabilities, it is of immense importance to collect and evaluate exact demographic data in marginalized subpopulations in the infectious disease context. Precisely because the COVID-19 pandemic isn’t affecting the population in the same way, epidemiological data on COVID-19 infection and mortality are needed to capture the scale and forms of the inequalities entrenched and magnified by the pandemic (Bhala et al. 2020; Chowkwanyun and Reed 2020). Thus, experts argue that policymakers and leaders of health systems need to release “racial and ethnic demographic data on COVID-19 infection and mortality” (Essien and Venkataramani 2020), including data on Indigenous people (UN 2020). These data should be collected and analysed, such as is done, for example, by the COVID Tracking Project (2020), which compiles data from U.S. health institutions.

However, important issues regarding power imbalances and vulnerability in relation to COVID-19 apps, which can complement the collection of demographic epidemiological data, warrant closer attention. As we will show here, a comprehensive debate about vulnerability in COVID-19 apps is necessary to prevent a pandemic-response, which would further exacerbate the vulnerability of the structurally marginalized subpopulations.

## Vulnerability in the COVID-19 Pandemic

In our discussion of vulnerability in the COVID-19 context, we use the taxonomy of vulnerability developed by Rogers et al. (2012). These scholars distinguish between *inherent* vulnerability, arising from one’s corporeality; *situational* vulnerability stemming from one’s personal, social, political, economic, or environmental situatedness as an individual or member of a group; and *pathogenic* vulnerability, emerging in sociopolitical contexts where a pre-existing vulnerability is multiplied by oppression or injustice (Rogers et al. 2012; Mackenzie et al. 2014).

In the COVID-19 pandemic, we observe that while all people are inherently vulnerable to the virus, racialized individuals are situationally more vulnerable to being infected by and dying from COVID-19 (Bhala et al. 2020; Benjamin 2020; Essien and Venkataramani 2020). Owing to structural disadvantage, racialized people are more likely to be in low-waged “essential” employment requiring them to commute to work and more commonly live in overcrowded housing which is difficult to self-isolate in, all of which makes them more susceptible to contracting the virus (Bhala et al. 2020; Benjamin 2020; Essien and Venkataramani 2020). Furthermore, this socioeconomic inequality translates to comorbidities, which make racialized people more prone to death from a COVID-19 infection.

While COVID-19 apps promise to decrease infection rates, if not designed and executed carefully, they risk making disadvantaged subpopulations pathogenically vulnerable. Scholarship on data surveillance (Benjamin 2019; Jefferson 2020) suggests that vulnerable subpopulations pay a higher price. Studies on digital technology such as facial recognition software and predictive policing demonstrate that racialized groups are typically subject to higher scrutiny and suffer greater negative consequences (Benjamin 2019; Poster 2019; Scannell 2019; Jefferson 2020). These include racial profiling and disproportionate policing, perpetuating the stigmatization and marginalization of already disadvantaged subpopulations. These risks ought to be taken seriously in debates on COVID-19 apps and related measures.

Since the outbreak of the COVID-19 pandemic, the world has seen a surge in anti-Asian stigmatization, including by political leaders (Nature 2020). There is also evidence of state authorities in different countries employing COVID-19 response tactics that disproportionately target structurally disadvantaged groups. In New York City, social distancing policing has led to the arrests of predominantly Black and Hispanic civilians (Southall 2020). In Australia the police have patrolled areas with high density of migrant subpopulations instead of predominantly white rich coronavirus hotspots such as Sydney beach neighbourhoods (Faruqi 2020; Blakkarly 2020). In several European countries, including Slovakia and Romania (Walker 2020; Korunovska and Jovanovic 2020), the police have cordoned off Roma neighbourhoods instead of quarantining infected individuals. The Roma inhabitants were reportedly prevented from accessing work outside of the settlements, which has further impoverished and endangered the subpopulation, with media outlets and NGOs reporting that individuals were cut off from water and medical supplies (Walker 2020; Korunovska and Jovanovic 2020). Cases like these have sparked a debate about COVID-19 as a “biopolitical reality” (Benjamin 2020) characterized by a racial double standard.

## The Need to Mitigate Vulnerability in COVID-19 Apps

In this context, for a comprehensive risk–benefit evaluation it is crucial to interrogate racial inequality and vulnerability in COVID-19 apps and related public health and safety measures. COVID-19 apps involve different ethical challenges pertaining to the particular type of technology and how the data are stored and who has access to them (Cho et al. 2020; Lucivero et al. 2020). In COVID-19 contact tracing apps, even if geolocation data are anonymized and self-reported, some schemes suggest the storage of the data in centralized databases run by state authorities. This can place situationally vulnerable groups at higher risk. Given that, in the past, digital surveillance has disadvantaged already marginalized groups and that current non-digital COVID-19 measures are biased, there is reason to worry that the precise digital localization of infection outbreaks and possible quarantine violations in marginalized areas such as racialized and/or migrant neighbourhoods might further the employment or intensification of disproportionate COVID-19 policing and the criminalization of the vulnerable. The potential use of COVID-19 apps to justify and normalize unjust interventions would be a clear example of measures grounded in broader structures of bias and oppression, which creates a fertile ground for pathogenic vulnerability in situationally vulnerable groups.

Proximity tracing technology, such as the PEPP-PT ( 2020), has been initially welcomed as a privacy-enhancing alternative, with the technology utilizing Bluetooth for contact tracing instead of geolocation data. However, beside a decentralized version of the technology which protects users by holding IDs locally on their devices (Troncoso et al. 2020), a centralized version is being developed which will involve data stored on servers controlled by state (health) authorities (Bock et al. 2020). This centralized version has generated criticism for potential access to data by governments (Bock et al. 2020; Ada Lovelace Institute 2020), which again raises concerns about how the data will be handled and with what implications for users, including marginalized subpopulations.

The evidence of racial inequality and vulnerability in the COVID-19 pandemic and social distancing policing should further the critique of centralized surveillance technologies which risk adding pathogenic to situational vulnerabilities in marginalized individuals. Furthermore, the risk that COVID-19 apps might exacerbate the vulnerability of racialized subpopulations can undermine public trust in COVID-19 apps. Racialized individuals’ lived experiences with racial discrimination (Benjamin 2019; Lentin 2020; Schaefer and Ballantyne 2020) can instil fear of further stigmatization and criminalization in the COVID-19 response and understandably deter them from their utilization and/or cause them to experience anxiety when under pressure to use these technologies. In order to meet the goal of infection control, it seems important to prioritize the most confidential and vulnerability mitigating COVID-19 technology—that is, technology without geolocation data tracing and with decentralized data storage and access.

## A Set of Ethical Values to Guide COVID-19 Apps

We agree with many of the ethics guidelines outlining values for the use of COVID-19 surveillance systems. These systems should respect privacy and confidentiality, be used for disease surveillance for the common good, and be scientifically sound, proportional, sustainable, and ethically justifiable (Morley et al. 2020; WHO 2020). We also agree that data collection on marginalized communities in the context of COVID-19 is necessary and should be upheld in order to reveal and better mitigate social vulnerabilities in pandemic responses and future pandemic planning. With respect to COVID-19 apps, the biopolitical reality permeated by structural inequalities calls for the explicit incorporation of values to guide the development and utilization of these technologies in order to mitigate social vulnerabilities (Enarson 2012; Chung and Hunt 2012) that could arise. The UNESCO (2020, 2) statement on COVID-19 emphasizes “our collective responsibilities for the protection of vulnerable persons and the need to avoid any form of stigmatization and discrimination, both verbal and physical.” Following on our discussion of vulnerability and broader debates on the ethical risks related to COVID-19 pandemic responses (Devakumar et al. 2020; Lucivero et al. 2020), it appears that beside the necessity of effectiveness and respect for privacy and confidentiality, COVID-19 apps ought to be explicitly grounded in values that include justice, equality, solidarity, and user-benefit. They should have robust oversight.

Values of justice, equity, and solidarity are important values in healthcare (Whitehead 1992; Krieger 1999; Thompson et al. 2006; Powers and Faden 2008; Venkatapuram 2011; Smith and Upshur 2019). Devakumar et al. (2020) emphasize that health protection does not only depend on effective universal healthcare systems but relies on social inclusion, justice, and solidarity. They argue that the absence of these values leads to the escalation of inequalities, scapegoating, and long-lasting discrimination, with broad negative public (health) outcomes. As such, these values are also central to the mitigation of situational and pathogenic vulnerability which stem from social contexts proliferated with inequality and oppression. The value of solidarity, in particular, emphasizes concern for structurally marginalized subpopulations (West-Oram and Buyx 2017; Jennings and Dawson 2015) and, as such, can guide public health measures to protect the most vulnerable.

Considering the risks that can stem from poorly designed and governed COVID-19 surveillance technology, it seems paramount that as a health technology, apps ought to provide health benefits to diverse target populations (Gasser et al. 2020), including marginalized groups. The value of beneficence reaches further beyond the basic healthcare requirement to avoid doing harm (Lipworth et al. 2018) and emphasizes the need for health interventions to improve public health outcomes equally. This is an important step, as a greater focus on benefit in vulnerable subpopulations can help mitigate pathogenic vulnerabilities (Hendl 2018) and increase health equity, which in turn increases population health for all.

Schaefer and Ballantyne (2020) argue that due to comorbidities, vulnerable people might have greater benefits from the use of COVID-19 apps. Yet, the authors acknowledge that the privacy risks to racialized people and their higher mortality rates place greater moral responsibility on privileged subpopulations to use surveillance technology. Nevertheless, all population groups should be able to use COVID-19 surveillance technology that respects their privacy without exacerbating their vulnerability via exposure to discriminatory “safety” measures.

In this regard, the integration of ethical values into the design and utilization of COVID-19 apps ought to be subject to robust oversight. Many have argued that, considering the serious risks involved, the current lack of regulation of COVID-19 surveillance technology is highly concerning and that strong governance and accompanying research are urgently needed (Lucivero et al. 2020; CIFAR 2020).

## Conclusion

Should current issues regarding efficacy, privacy, data protection, and concerns regarding vulnerability be resolved, then COVID-19 apps could be viable tools for the suppression of the pandemic. Underlying their design and implementation should, however, be the value of justice in healthcare, understood more holistically than as a matter of unequal distribution of smartphones. If apps are promoted as an integral part of the COVID-19 pandemic response, then this should be done with a clear and explicit commitment to values of health equity, non-discrimination, and solidarity with vulnerable subpopulations.

Furthermore, if COVID-19 apps are implemented, then it will be important to require developers and implementers to outline strategies to mitigate social vulnerabilities. Simultaneously, accompanying research is warranted inquiring into a potential increase of biased measures related to the use of apps, such as qualitative studies interviewing marginalized groups on how they value the technology and whether they experience any negative effects.

Finally, some (Floridi 2020; Gasser et al. 2020) have emphasized that digital infectious disease surveillance and related measures need to be ceased at the end of the pandemic. Yet, the COVID-19 pandemic might not end anytime soon, and a general global preparedness for other pandemics is necessary (Smith and Silva 2015; Rahimi and Abadi 2020). Thus, it is even more important to collect data about infectious diseases in all subpopulations and ensure that pandemic surveillance technology and public health measures are grounded in robust ethical values, including justice and a commitment to mitigate vulnerability of the most disadvantaged and at-risk individuals and subpopulations.
